# Supplementary material for: Association between muscle strength and depression in a cohort of young adults
Source: PLoS One. 2024 May 31;19(5):e0303925. doi: 10.1371/journal.pone.0303925 (PMC11142448; doi:10.1371/journal.pone.0303925)
Supplement: S1 Table — (DOCX) [file pone.0303925.s001.docx]

**S1 Table.** **Overview of** **descriptive statistics from muscle strength according to tertiles.**

| **Variable** | **Tertiles** | ***n*** | **Median** | **Mean** | **SD** | **Skewness** | **Kurtosis** | **Min** | **Max** | **25th**  **percentile** | **50th**  **percentile** | **75th**  **percentile** |
| --- | --- | --- | --- | --- | --- | --- | --- | --- | --- | --- | --- | --- |
|  |  |  |  |  |  |  |  |  |  |  |  |  |
| EXT_R | T1 | 149 | 1.60 | 1.54 | 0.21 | -2.12 | 6.68 | 0.49 | 1.75 | 1.45 | 1.60 | 1.68 |
|  | T2 | 622 | 2.22 | 2.22 | 0.25 | 0.05 | -1.11 | 1.76 | 2.67 | 2.01 | 2.22 | 2.41 |
|  | T3 | 137 | 2.88 | 2.94 | 0.27 | 4.33 | 31.67 | 2.68 | 5.14 | 2.78 | 2.88 | 3.01 |
| EXT_L | T1 | 131 | 1.55 | 1.47 | 0.26 | -2.08 | 6.14 | 0.24 | 1.72 | 1.38 | 1.55 | 1.65 |
|  | T2 | 651 | 2.19 | 2.18 | 0.26 | -0.03 | -1.16 | 1.73 | 2.63 | 1.96 | 2.19 | 2.41 |
|  | T3 | 127 | 2.82 | 2.87 | 0.28 | 4.01 | 25.99 | 2.64 | 4.98 | 2.70 | 2.82 | 2.96 |
| FLEX_L | T1 | 148 | 0.80 | 0.77 | 0.13 | -2.59 | 10.48 | 0.02 | 0.90 | 0.72 | 0.80 | 0.85 |
|  | T2 | 609 | 1.18 | 1.19 | 0.15 | 0.10 | -0.96 | 0.91 | 1.47 | 1.07 | 1.18 | 1.31 |
|  | T3 | 152 | 1.59 | 1.63 | 0.14 | 1.97 | 8.09 | 1.48 | 2.46 | 1.52 | 1.59 | 1.70 |
| FLEX_R | T1 | 141 | 0.79 | 0.76 | 0.15 | -2.73 | 8.87 | 0.04 | 0.89 | 0.72 | 0.79 | 0.85 |
|  | T2 | 617 | 1.17 | 1.17 | 0.15 | 0.09 | -1.01 | 0.90 | 1.47 | 1.05 | 1.17 | 1.30 |
|  | T3 | 150 | 1.60 | 1.64 | 0.15 | 2.84 | 14.48 | 1.48 | 2.68 | 1.53 | 1.60 | 1.69 |
| FLEX+EXT_L | T1 | 132 | 2.38 | 2.27 | 0.38 | -2.38 | 8.28 | 0.26 | 2.66 | 2.15 | 2.38 | 2.51 |
|  | T2 | 645 | 3.39 | 3.37 | 0.39 | -0.02 | -1.08 | 2.67 | 4.07 | 3.04 | 3.39 | 3.69 |
|  | T3 | 132 | 4.34 | 4.44 | 0.39 | 3.80 | 24.80 | 4.08 | 7.44 | 4.19 | 4.34 | 4.58 |
| FLEX+EXT_R | T1 | 135 | -1.37 | -1.50 | 0.49 | -2.44 | 9.18 | -4.03 | -1.01 | -1.67 | -1.37 | -1.18 |
|  | T2 | 638 | -0.03 | -0.02 | 0.55 | 0.04 | -1.12 | -1.00 | 0.99 | -0.51 | -0.03 | 0.42 |
|  | T3 | 135 | 1.47 | 1.57 | 0.59 | 4.02 | 27.93 | 1.00 | 6.23 | 1.18 | 1.47 | 1.79 |
| EXT_R+L | T1 | 139 | 3.18 | 3.04 | 0.43 | -2.23 | 7.47 | 0.73 | 3.49 | 2.87 | 3.18 | 3.34 |
|  | T2 | 637 | 4.38 | 4.40 | 0.48 | 0.04 | -1.16 | 3.52 | 5.27 | 3.98 | 4.38 | 4.83 |
|  | T3 | 133 | 5.65 | 5.75 | 0.54 | 4.38 | 32.18 | 5.28 | 10.12 | 5.39 | 5.65 | 5.93 |
| FLEX_R+L | T1 | 145 | 1.60 | 1.54 | 0.26 | -2.87 | 12.72 | 0.10 | 1.81 | 1.46 | 1.60 | 1.70 |
|  | T2 | 616 | 2.36 | 2.36 | 0.30 | 0.11 | -0.99 | 1.82 | 2.93 | 2.12 | 2.36 | 2.60 |
|  | T3 | 148 | 3.19 | 3.24 | 0.27 | 2.82 | 15.41 | 2.94 | 5.14 | 3.05 | 3.19 | 3.35 |

EXT_R, muscle strength right knee extensors. FLEX_L, muscle strength left knee flexors. FLEX_R, muscle strength right knee flexors. EXT_L, muscle strength left knee extensors. FLEX+EXT_L, muscle strength thigh muscles left leg. FLEX+EXT_R, muscle strength thigh muscles right leg. EXT_R+L, muscle strength right and left extensors. FLEX_R+L, muscle strength right and left flexors. T*_i_* tertiles.
